# En bloc resection of extra-peritoneal soft tissue neoplasms incorporating a type III internal hemipelvectomy: a novel approach

**DOI:** 10.1186/1477-7819-10-222

**Published:** 2012-10-25

**Authors:** Sanjay S Reddy, Norman D Bloom

**Affiliations:** 1 Fox Chase Cancer Center, Department of Surgical Oncology, 333 Cottman Avenue, Philadelphia, PA, 19102, USA; 2 Beth Israel Medical Center, Department of Surgery, 1st avenue at 16th street, New York, NY, 10003, USA

## Abstract

**Background:**

A type III hemipelvectomy has been utilized for the resection of tumors arising from the superior or inferior pubic rami.

**Methods:**

In eight patients, we incorporated a type III internal hemipelvectomy to achieve an en bloc R0 resection for tumors extending through the obturator foramen or into the ischiorectal fossa. The pelvic ring was reconstructed utilizing marlex mesh. This allowed for pelvic stability and abdominal wall reconstruction with obliteration of the obturator space to prevent herniations.

**Results:**

All eight patients had an R0 resection with an overall survival of 88% and with average follow up of 9.5 years. Functional evaluation utilizing the Enneking classification system, which evaluates motion, pain, stability and strength of the affected extremity, revealed a 62% excellent result and a 37% good result. No significant complications were associated with the operative procedure. Marlex mesh reconstruction provided pelvic stability and eliminated all hernial defects.

**Conclusion:**

The superior and inferior pubic rami provide a barrier to a resection for tumors that arise in the extra-peritoneal pelvis extending through the obturator foramen or ischiorectal fossa. Incorporating a type III internal hemipelvectomy with a simple marlex mesh reconstruction allows for complete tumor resection without functional compromise, acute infectious issues, obturator or abdominal hernia defects.

## Introduction

A novel approach to the en bloc resection of extra-peritoneal soft tissue neoplasms, incorporating a type III internal hemipelvectomy, to achieve clear surgical margins, was performed in eight patients.

The use of a type III internal hemipelvectomy in the resection of primary bone tumors has been widely employed. Type III resections, as classified by Enneking and Dunham involves resection of the superior and inferior pubic rami and obturator foramen [1]. The use of type III internal hemipelvectomy for osseous lesions is not common. In two large series of internal hemipelvectomies, type III has been performed in three out of thirty patients (10%) [2], and in four out of fifty-eight patients (7%) [3]. In this series of patients we have extended the indication for this procedure to soft tissue tumors arising in the extraperitoneal pelvis with extension through the obturator foramen into the adductor group, or into the ischiorectal fossa. This is a rare presentation for these tumors and any standard operative approach for a complete resection would lead to a violation of the tumor, as the central bony pelvis provides a barrier to an en bloc resection.

A type III hemipelvectomy is not without its associated complications, which include vascular, bladder and urethral injuries, as well as infections and wound issues related to the groin incision [4]. No reconstruction of the pelvic ring has been used in many cases of type III internal hemipelvectomy, however, allografts and autografts are still utilized [5].

## Methods

From 1987 to 2011 eight patients presented to NDB with soft-tissue tumors of the extra-peritoneal pelvis extending through the obturator foramen or into the ischiorectal fossa. We employed an operative technique in all of these patients that incorporated a central pelvic bone resection (type III internal hemipelvectomy), to achieve an en bloc resection and clear surgical margins. This technique was employed because these tumors both extended into and through the obturator foramen into the adductor group, or below the ischium.

**Figure 1:**
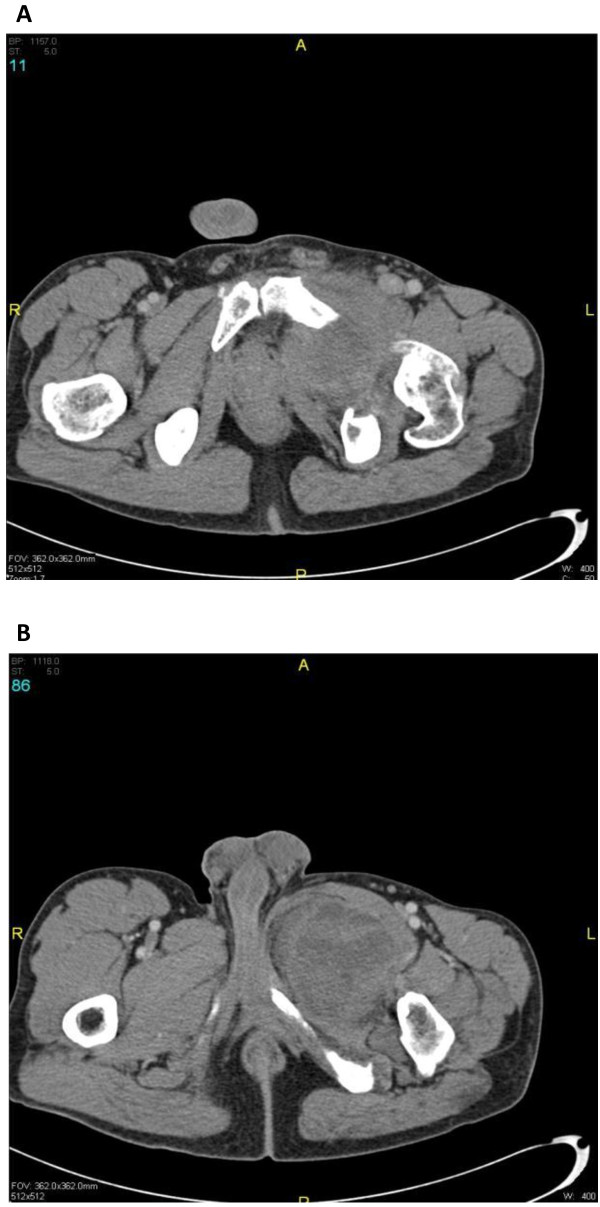
Pre-Operative CT Scan: a malignant peripheral nerve sheath tumor with extension through the obturator foramen in a 36 year-old man with Von Recklinghausen disease.

Two cases illustrate these presentations and the surgical techniques involved. As an example of a tumor extending through the obturator foramen, a 36 year-old male presented with a slowly growing mass in his left upper thigh for six months. He had an unrecognized history significant for Von Recklinghausen’s disease, and manifested this with several neurofibromas throughout his body, in addition to scattered café au lait spots. His main complaints were of difficulties walking secondary to mass effect, and pain in his left thigh. Pre-operative imaging by computer tomography (CT) was significant for a dominant heterogeneous soft-tissue mass in the left inguinal region causing bony remodeling (Figure 1).

**Figure 2:**
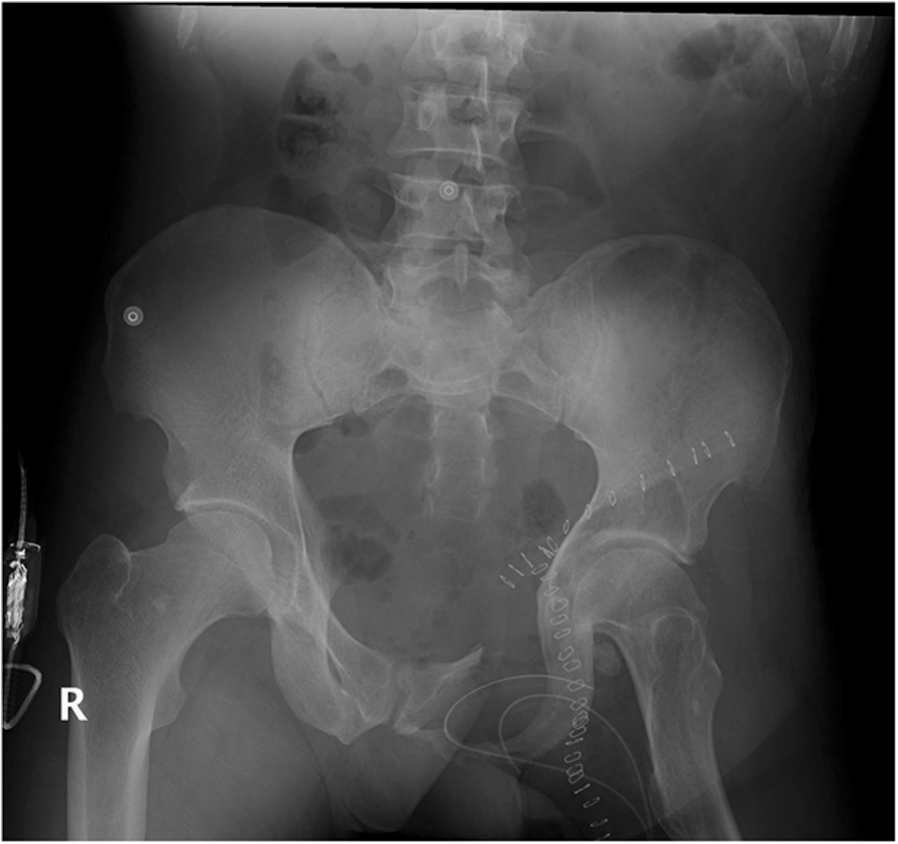
Post-resection radiograph depicts the bony defect and the ilio-inguinal incision.

Intra-operatively, there was evidence of a large lesion occupying the adductor group of the left thigh with direct extension through the obturator foramen, with intra-pelvic tumor extension along the obturator nerve. The patient underwent wide resection of the lesion encompassing the adductor muscle group in continuity with the en bloc resection of the superior pubic rami, the entire obturator foramen, and the intra-pelvic tumor that extended along the obturator nerve. Once the specimen was removed en bloc, the reconstruction of the pelvic defect was undertaken (Figure 2). 

After an uneventful hospital stay, he was discharged on post-operative day 7. He has had follow-up at three-, six- and nine-month intervals, and has been doing well. He reports occasional pain, however, is able to walk without assistance, has a well-healed incision with no signs of infection, and no evidence of a hernia. He has returned full-time to his occupation as an excavator.

**Figure 3:**
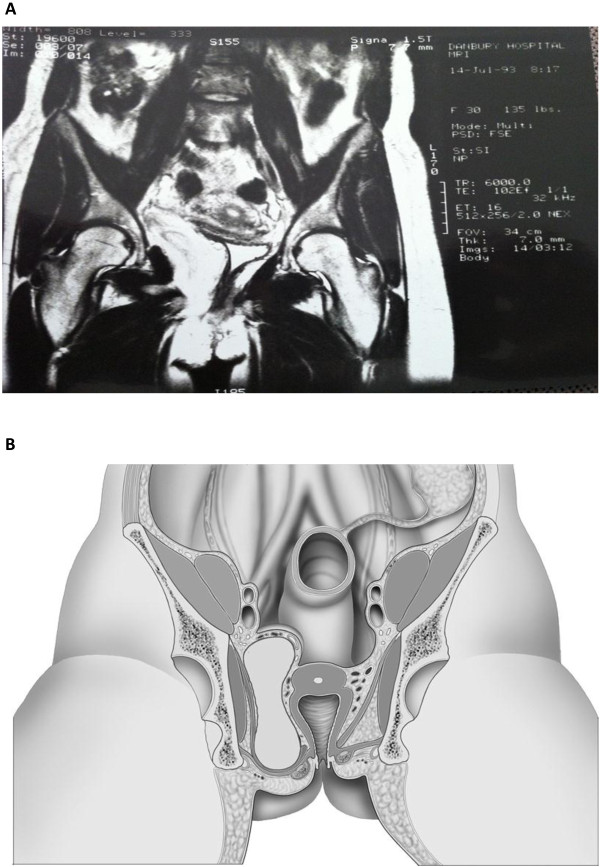
**(A) Angiomyxoma on MRI demonstrating tumor extension into the ischiorectal fossa.** (**B**) Illustrative diagram demonstrating tumor extension into the ischiorectal fossa.

The second case is a 31 year-old woman who presented with a mass bulging into her ischiorectal fossa. Eight months earlier, she had undergone incomplete resection of an angiomyxoma via a lower midline abdominal incision. On clinical examination she had an obvious mass protruding into the ischiorectal fossa adherent to the lateral rectal and vaginal walls. Magnetic resonance imaging (MRI) revealed a large mass occupying the pelvis on the right with direct extension into the perineum (Figure 3).

**Figure 4:**
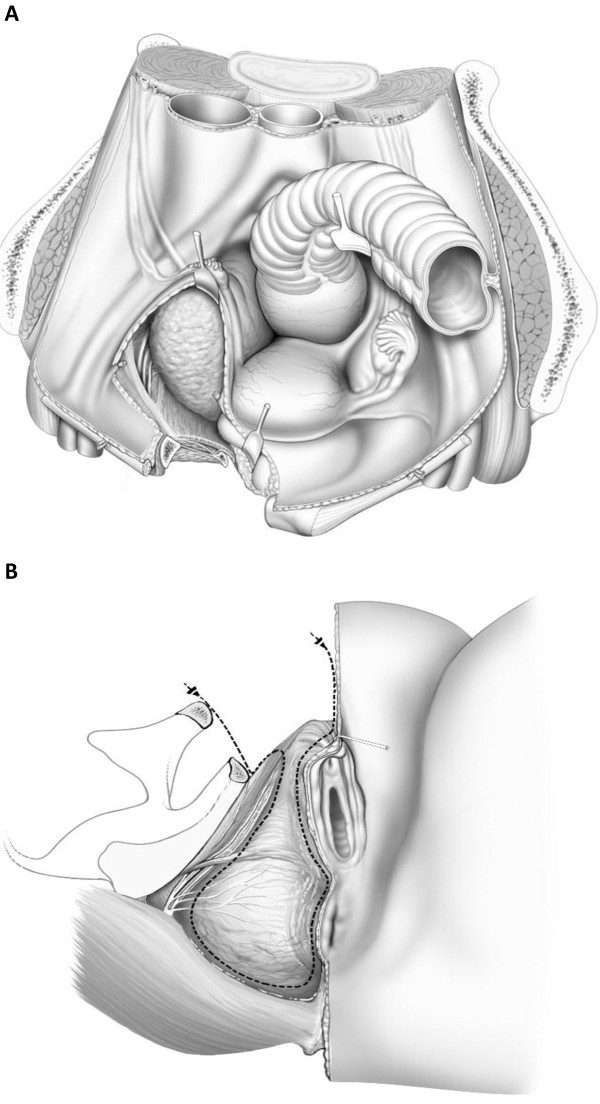
Illustrative diagram demonstrating the resected superior and inferior pubic rami, allowing exposure of the tumor in the extra-peritoneal pelvis and ischiorectal fossa.

Upon exploration, it was evident that the mass occupied the extra-peritoneal pelvis with extension into the perineum and ischiorectal fossa. The symphysis pubis was divided, and a sub-periosteal resection of the superior and inferior pubic rami to the middle of the obturator foramen was performed. With the exposure given from this maneuver, the perineum and the extra-peritoneal pelvis, along with the recurrent mass, was resected en bloc with the levators and the right lateral wall of the vagina in continuity (Figure 4). The patient had an unremarkable post-operative course and began ambulating within 48 hours. She received post-operative external beam radiotherapy, and remained disease-free after 20 years of follow-up. Three years after the resection she ran a marathon.

**Figure 5:**
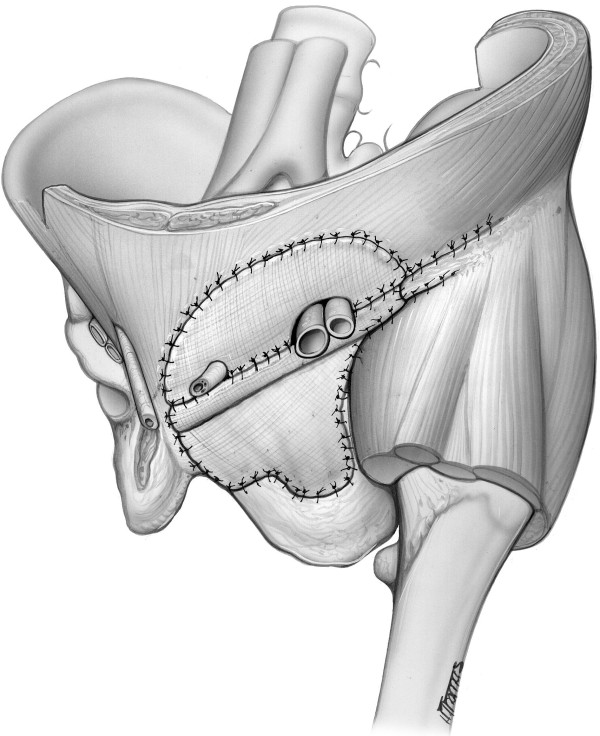
Illustrative diagram demonstrating a marlex mesh reconstruction of the bony pelvic ring and the abdominal wall.

In our series of patients, the technique chosen to reconstruct the pelvic ring involves the use of Marlex mesh (CD Bard). A single sheet of mesh was taken, and the anterior edge folded upon itself with 1-cm overlap. The primary structures to which the mesh was secured were the symphysis pubis medially and the anterior superior iliac spine laterally. Holes were then created in the mesh to allow passage of the femoral vessels and the cord structures. Next, a second piece of marlex mesh was utilized, and secured superiorly to the rectus abdominis muscles, and inferiorly to the superior border of the primary mesh (Figure 5). The adductor muscles were similary attached.

## Results

Five patients had malignant tumors, the most common being a malignant peripheral nerve sheath tumor and three patients had locally aggressive benign tumors, two of which were desmoids and one angiomyxoma. The age of the patients ranged from 26 years to 58 years of age, six were male and the remaining two female. The average patient follow-up was 9.5 years. Seven of the eight patients (88%) had follow-up beyond 5 years. One patient in the series died of metastatic disease. Seven of eight patients received post-operative radiation therapy; one patient received intra-operative radiation in addition to post-operative radiation. The single patient that did not receive radiotherapy presented with a locally recurrent desmoid tumor, for which repeat resection was undertaken. One patient developed a superficial wound infection, and one developed significant leg lymphedema after radiation therapy (Table 1).

**Table 1:** Type III internal hemipelvectomy database

**Case**	**Sex**	**Age (yr)**	**Site**	**Histology**	**Complication**	**Result therapy**	**Adjuvant**	**F/U (yr)**
1	M	58	Groin	MFH	Skin necrosis	Good	+RT	15 NED
2	M	26	Groin	MPNS	None	Excellent	+RT	13 NED
3	M	29	Pelvis	MPNS	None	Excellent	+RT	14 NED
4	M	55	Pelvis	Liposarcoma	Death	Good	+RT	5 DWD
5	M	36	Pelvis	MPNS	None	Excellent	+RT	1 NED
6	M	26	Pelvis	Desmoid	None	Excellent	-RT	5 NED
7	F	35	Pelvis	Desmoid	Leg edema	Good	+IORT/RT	5 NED
8	F	31	Pelvis	Angiomyxoma	None	Excellent	+RT	18 NED

MFH = Malignant Fibrous Histiocytoma; MPNS = Malignant Peripheral Nerve Sheath Tumor; IORT= intra-operative radiotherapy.

Patients undergoing limb salvage surgery for bone and soft-tissue sarcoma of the extremities can experience significant physical disability as a result of life-preserving treatment. The patients in our series all had good or excellent results. This objective evaluation was achieved using the modified Enneking classification for functional outcomes. The primary factors were motion, pain, stability/deformity, strength, emotional acceptance/functional ability and complications [6]. Depending on a point system, patients were labeled as having excellent, good, fair or poor outcomes. For excellent, five of the six primary factors must rate excellent. The sixth may be good, fair or poor. For a rating of good, five of six factors must be good or better with one fair or poor. The detailed classification of the two cases described here is shown (Table 2); the remaining six patients were similarly evaluated.

**Table 2:** Enneking’s classification of two patients

**Outcome**	**Case 1**	**Case 2**
Motion	E	E
Pain	E	E
Stability/deformity	E	E
Strength	G	E
Emotional acceptance/function	E	G
Complication	E	E
Overall	E	E

E, excellent; G, good; F, fair; P, poor.

## Discussion

Tumors of the extraperitoneal pelvis with extension through the obturator foramen, or into the ischiorectal fossa, present a surgical challenge to accomplish an en bloc resection. The intact pelvic ring prevents direct access for tumor resection. We have employed a surgical technique incorporating a type III internal hemipelvectomy to achieve an en bloc resection for tumors arising in this region.

The internal hemipelvectomy has been widely employed in lieu of a traditional hemipelvectomy for malignant tumors arising from the pelvic bone. In 1978 Enneking classified the internal hemipelvectomy into three types. Type I involves resection of the ilium, type II involves a periacetabular resection, and type III involves resection of the superior and inferior pubic rami. In our series of eight patients, a type III internal hemipelvectomy was performed to achieve an en bloc resection of the primary tumor, despite the lack of bone involvement. This approach allowed direct access to, and complete resection of tumors that extended through or under the bony barrier.

A simple technique utilizing Marlex mesh was used to reconstruct the bony defect and prevent obturator or ischiorectal herniations. Marlex mesh was utilized because it had been used extensively for the repair of primary or recurrent inguinal hernias. In a series of 3,000 patients there was an insignificant infection rate of 0.2% [7]. Marlex mesh has also been used to repair extensive chest wall and abdominal wall defects following tumor resection [8], or in abdominal trauma with intra-abdominal sepsis [9]. Its ability to stabilize the chest wall after major resections encouraged us to employ this material to restore and stabilize the pelvis. By re-attaching the abdominal and extremity musculature to its insertions and origins, all dead space was obliterated, thus eliminating one major contributor to deep space infections.

More sophisticated reconstructive techniques employing autografts or allografts have been utilized for type III reconstructions. Based on 206 patients who underwent pelvic resection at Massachusetts General (of whom 41 had allografts), Mankin reported a 20% infection rate in patients undergoing pelvic allografts [10]. The majority of the 41 patients had undergone a more extensive pelvic resection but there were several allografts type III reconstructions included in this series.

Non-union of pelvic allografts is another potential complication, as is graft failure [11]. In our series of eight patients there was only one superficial wound infection, and no separation of the mesh from either its bone or muscle attachments. As such, it provides an excellent simple reconstructive technique to eliminate the obturator hernial defect, reconstitute the pelvic defect and reduce complications due to infection.

This operative approach allows for good to excellent functional outcomes without compromising tumor resection.

## Competing interest

The authors declare that they have no competing interest.

## Authors’ contributions

All patients presented to NDB for operative planning. SSR conducted the retrospective analysis and drafted the manuscript. All authors read and approved the final manuscript.
